# Call for equitable hearing healthcare provision: an exploration of Māori adults’ perspectives and lived experiences of ear and hearing healthcare services in Te Waipounamu

**DOI:** 10.1080/03036758.2024.2417665

**Published:** 2024-10-23

**Authors:** Tare Lowe, Jennifer Smith, Alehandrea Raiha Manuel, Greg A. O’Beirne

**Affiliations:** a School of Psychology Speech and Hearing, Te Whare Wānanga o Waitaha|The University of Canterbury, Ōtautahi, New Zealand; b Child Well-being Research Institute, Te Whare Wānanga o Waitaha|The University of Canterbury, Ōtautahi, New Zealand; c School of Population Health, Faculty of Medical and Health Sciences, Waipapa Taumata Rau|The University of Auckland, Tāmaki Makaurau, New Zealand; d Eisdell Moore Centre, Waipapa Taumata Rau|The University of Auckland, Tāmaki Makaurau, New Zealand

**Keywords:** Kaupapa Māori research, indigenous languages, te reo Māori, audiology, digit triplet test, hearing healthcare, Māori health

## Abstract

Māori adults are over-represented in self-reported hearing loss statistics in Aotearoa, yet they are currently underserved in hearing healthcare services that were designed using one-size-fits-all Eurocentric ideologies and models of health and disability. Unassisted hearing loss has been linked to increased rates of depression, social isolation, communication issues, early retirement, and cognitive decline, as well as a decreased ability to engage with cultural practices. An area that requires further research is the exploration of Māori adults’ perspectives and experiences of hearing healthcare. This article uses a Kaupapa Māori lens to bring forward Māori narratives and expands on previous research of a te reo Māori hearing screening test. The article shares findings from interviews with Māori adults, exploring both their perspectives and lived experiences of ear and hearing healthcare and te reo hearing assessments. It highlights the barriers that exist for Māori when accessing hearing healthcare in Te Waipounamu (The South Island of Aotearoa) and the desire to see transformative change in the hearing healthcare landscape.

## Abstract (es)

Glossary of Māori words:

Ahitereiria ki te Hauāuru: Western Australia; Aotearoa: North Island of New Zealand – now used as the Māori word for New Zealand; aroha ki te tangata: a respect for the people; hauora: health, wellbeing; ia: te reo Māori pronoun for everyone; iwi: tribe; kai: food; kai tahu/ngai tahu: tribal group of the South Island; kanohi kitea: the seen face; kanohi-ki-te-kanohi: face-to-face; kaua e takahia te mana o te tangata: do not trample over the mana of people; kaumātua: Māori elder/s; kaupapa: topic, policy, thing of discussion; kaupapa Māori: a philosophical approach using Māori knowledge and values; Māori approach; kia māhaki: be humble; koha: gift, present, offering; kōrero: narratives and prose; kūmara: sweet potato; kupu: word, vocabulary, saying; manaaki ki te tangata: share and host people, be generous; mana motuhake: self-determination, authority and capacity to be autonomous; Māori: Indigenous people of Aotearoa New Zealand; mātauranga Māori: Indigenous Māori knowledge; Ngāpuhi: a tribal group situated in the top of the North Island; Ngāti Porou: a tribal group of the East Coast of the North Island; Ngāti Whātua: tribal group between the Hokianga Harbour and Tāmaki; ōritetanga: equity, equal outcomes; Ōtautahi: traditional name known now as Christchurch; Pākehā: European New Zealander, non-Māori; Tangata Tiriti: people of Te Tiriti, referring to all non-Māori citizens and residents of Aotearoa; Tangata Whenua: people of the land, Indigenous peoples, local people, hosts; Tauiwi: foreign people, non-Māori; te ao Māori: the Māori world; te reo Māori (te reo): the Māori language, Indigenous language of Aotearoa; Te Tiriti o Waitangi: signed in 1840 this is the Māori language version of an agreement between two internationally recognised sovereign nations, Māori, as tāngata whenua, and the British Crown; Te Waipounamu: the South Island of Aotearoa; Te Whakamātautau Whakarongo o Aotearoa: Te Reo Māori Digit Triplet Test, TRMDTT; tikanga: Māori customs, procedures, protocols, practices, system of values; tino rangatiratanga: sovereignty and self-determination; titiro, whakarongo … kōrero: look, listen … speak; tuākana-teina: older-younger; tuatoru: third; whakaaro: thoughts, opinions, and considerations; whakamā: shame, embarrassment, guilt; whakawhanaungatanga: an indigenous process of creating relational connection; whānau: immediate & extended family and friends.

## Introduction

In Aotearoa New Zealand (Aotearoa), Māori are disproportionately impacted by hearing loss and have limited access to culturally appropriate assessments or services (Crisp 2010; Lowe 2023; Manuel et al. 2024). Such realisations are underpinned by inequities in the socioeconomic determinants of health due to the ongoing implications of colonisation and the consequence of loss of land, cultural connection, te reo Māori [te reo] (the Māori language) as well as political disempowerment and social marginalisation (Anderson et al. 2006; Moewaka Barnes and McCreanor 2019). Although there was once a push from government to improve service delivery of hearing healthcare to Māori, limited work has been done in this area for the past 30 years to reduce inequities in the hearing healthcare system (The Review Team, 1989).

The use of patient-directed te reo in primary care settings has been shown to have a direct impact on patients’ perception of quality of care (Pitama et al. 2011). Thus, the development of audiological assessments in te reo is vital to improving audiological service delivery and ensuring culturally safe hearing healthcare is available in Aotearoa. This overarching goal forms the focus of this research and its companion papers’ Dawson et al. (2024) and Manuel et al. (2024).

Hearing screening tests such as the digit triplet test are popular worldwide as they have been shown to increase early identification and promote awareness of hearing loss (Smits and Houtgast 2005; Jansen et al. 2010). A digit triplet test is not currently part of clinical protocol in Aotearoa, although one that has been developed in New Zealand English is available online to provide an easily accessible and objective test to raise awareness about hearing loss (King, 2011). ‘Te Whakamātautau Whakarongo o Aotearoa’ is a Te Reo Māori Digit Triplet Test (TRMDTT), first developed in 2011/2012 (Murray, 2012; Bowden, 2013). Dawson et al. (2024) recently contributed to the validation of TRMDTT, exploring more effective methodological approaches for the development of te reo audiological assessment tools.

The development of audiological assessments in te reo has not been explored in the literature beyond TRMDTT, and as that has yet to be released, there are currently no publicly available validated audiological assessments in te reo. This research extends the work of Murray (2012), Bowden (2013), and Dawson et al. (2024), by undertaking a series of interviews with the participants of the latter study, using reflexive thematic analysis (Braun and Clarke, 2021) to explore their attitudes towards the place of a TRMDTT, and themes relating to their perspectives and lived experience of hearing loss and ear and hearing healthcare in Aotearoa.

## Methodology

Hearing health research that is conducted ‘on’ Indigenous peoples often maintains systems of institutionalised racism which produce and perpetuate systemic hearing health inequities (Huria et al. 2019; Haitana et al. 2020; Smith 2021). To address this, such research approaches need to bring forward Indigenous expertise, priorities, and knowledge systems (Huria et al. 2019). This current research is underpinned by Kaupapa Māori Research (KMR).

KMR challenges neo-colonial dominance often seen in research by valuing te ao Māori (the Māori world), mātauranga Māori (Indigenous Māori knowledge), whakaaro (thoughts, opinions, and considerations) and tikanga Māori (Māori customs, procedures, protocols, practices, system of values) in research (Bishop 1998; Cram et al. 2004; Walker et al. 2006; Pihama 2010; Smith et al. 2012). Smith (2021) outlines KMR research values, including the concepts of aroha ki te tangata (a respect for the people); kanohi kitea (the seen face); titiro, whakarongo … kōrero (look, listen … speak); ‘kia māhaki’ (be humble); ‘manaaki ki te tangata’ (share and host people, be generous); and ‘kaua e takahia te mana o te tangata’ (do not trample over the mana of people). In this research, we honour Māori beliefs, values, experiences and worldviews through this critical ‘by, with, and for Māori’ KMR approach.

The need for hearing healthcare research to be in partnership with Māori, where Māori voices are heard and put forward is important. This Māori-led KMR prioritises actions that enact mana motuhake (self-determination, authority and capacity to be autonomous) and protects the rights of Māori to exercise tino rangatiratanga (sovereignty, self-determination). Mana motuhake and tino rangatiratanga are related terms, although Hawksley and Howson (2011) claim the former implies a stronger connection with land and political autonomy than the latter. In the mitigation of power imbalances, Māori who were part of this research have been positioned as research partners through this kaupapa (topic of discussion), reinforcing their importance as experts in their perspectives and experiences of ear and hearing healthcare. This enhances the transformative potential of the research and helps to ensure that the knowledge and information are retained within the community.

Researchers should also respond and think critically about their influence in processes and relationships within KMR (Smith, 2021). The reflective practice of our individual positionings provides opportunities to consider and acknowledge the wider societal constructs and power dynamics within research. It also provides space for Māori values and knowledge to be considered within this research and how it applies to the future of hearing healthcare in Aotearoa. Each of our positionings within this research team is presented below:

Tare: Ko Kāi Tahu te iwi. E noho ana au ki Ōtautahi. I am a Māori audiologist and emerging Kaupapa Māori researcher. I have lived experiences of chronic middle ear issues and whānau members (immediate and extended family and friends) who live with hearing loss. It is these experiences which motivate me to create better hearing healthcare for Māori. I would like to acknowledge firstly Aleh, Jen, Greg and James, this would not have been possible without your knowledge, mentorship, support and encouragement and importantly the research partners I had the honour of working with, what I learnt from our kōrero (narratives and prose) has shaped the audiologist I am today. Kāore he mutunga o tēnei mea, te ako. There is no such thing as an end to learning.

Jennifer: Nō Ngāti Whātua, nō Ngāpuhi te wahine nei. I am a hard-of-hearing, second-language speaker of te reo Māori, an indigenous woman. I come from a background of education but rarely did my educational career allow me to learn more about my hearing, or lack thereof, in ways that reflected my identity, my Māoritanga.

Alehandrea (she/her/ia): Ko Ngāti Porou te iwi. Nō Philippines (Ilocos) tōku māmā. Being part of this kaupapa and a collective team has filled my ngākau, and the dream of seeing an equitable future in ear and hearing healthcare seems closer. Seldom do whānau Māori have the choice to become a Māori hearing healthcare worker. It has been a privilege watching our kūmara (sweet potato) vine grow with more Māori audiologists including that of Tare. Kia rongo a Puku i te mamae. It has been a beautiful healing journey with laughter.

Greg: Ko Tauiwi te iwi. He Pākehā au nō Ahitereiria ki te Hauāuru. I am fortunate to have never experienced the discrimination of trying to navigate a healthcare system that was not designed for me and remain grateful to our research partners for sharing their voices and lived experiences.

## Recruitment

Researchers should also respond and think critically about their influence in processes and relationships Purposive sampling was used to select eight research partners from those who previously participated in the validation of TRMDTT undertaken by Dawson et al. (2024) the year prior. These were Māori adults over the age of 18 who either did or did not speak te reo. Not all who participated in the validation study were hard-of-hearing so those who were identified as having lived experiences of hearing loss were contacted first. Due to the limited number of potential research partners personally experiencing hearing loss, lived experience was extended to include whānau experiences of hearing loss and ear and hearing healthcare services.

## Procedure

A semi-structured interview design was used for this project to align with a KMR approach. The interview schedule included questions around perspectives and experiences of hearing loss, completing TRMDTT, and utilisation of available ear and hearing healthcare services. Within the interviews space was open for research partners to express their individual experiences and perspectives and for the lead researcher (TL) to ask follow-up questions based on the responses.

All research partners gave informed consent, chose a time and place they felt comfortable with for the interview, and were able to bring a support person. Semi-structured interviews with eight research partners were organised to be kanohi-ki-te-kanohi (face-to-face) or online via video conference. One research partner opted for an online video conference. The interviews were digitally recorded and transcribed using commercial transcription software. Research partners were provided the option to review a copy of their transcript. Each research partner received a kai (food) bag and koha (gift/custom of gifting) which, through the spirit of reciprocity, acknowledged their contributions to this kaupapa.

## Data analysis

Researchers should also respond and think critically about their influence in processes and relationships Reflexive thematic analysis (RTA) was conducted using the interview transcripts, which were coded using NVivo data analysis software (Lumivero, Denver, CO, USA). A key element of RTA is acknowledging the subjective skills of the researcher and identifying and articulating their theoretical assumptions through reflexivity (Braun and Clarke 2021). RTA was used within a constructionist framework and undertaken using Braun and Clarke’s six-step recursive process (Braun and Clarke, 2006, 2021; Burr 2015; Byrne 2022).

All interviews were coded by the lead researcher (TL). AM, JS, and GO coded one of the transcripts to cross-check the initial coding framework. Interpretation of data was viewed through a kaupapa Māori lens, and in alignment with KMR principles and values. This ensured that the mana of research partners was upheld. Several meetings occurred to cross-validate and refine codes and themes within a tuakana-teina (older-younger) mentorship and support space, and to ensure the codes fit within a KMR approach. Thematic maps were used as a tool to develop, review, and refine the study themes and sub-themes.

## Ethics

Consultation with iwi through the Ngāi Tahu Consultation and Engagement Group at the University of Canterbury occurred as part of the ethics approval process and was approved in March 2022. Prior to commencing any interviews, this project obtained approval from the University of Canterbury Te Whare Wānanga o Waitaha Human Research Ethics Committee (HREC) (Ref. no.: HREC 2022/09/LR).

## Results

A total of eight research partners (self-identified as four male, four female) were interviewed – three had personal experiences of hearing loss and five had a close whānau (immediate and extended family network) member or friend who experienced hearing loss. Of the eight, three also chose to comment on personal experiences of ear and hearing healthcare services beyond audiology which included Ear, Nose, Throat specialist or ear nurse services. The youngest research partner was in their 30s and the oldest in their 70s. All research partners resided in Ōtautahi Christchurch.

From the RTA, two primary themes *‘That’s not for me’* and ‘*You can’t go wrong with more tikanga’* were identified, each with sub-themes which build the picture of research partners’ perspectives and experiences of ear and hearing healthcare in Te Waipounamu (the South Island of Aotearoa). Quotes from research partners using pseudonyms are presented in Table 1.

**Table 1.** Selected research partner responses during semi-structured interviews grouped into subthemes.

Subtheme		Quotes
Poor access to hearing loss education	**1**	‘I’ve seen whānau that I work with in the community not realise that they have a hearing impairment or they have trouble hearing because they think it’s something else’ *(Maia)*
Cost as a barrier to access	**2**	‘if the outcome is the worst outcome and he needs a hearing aid, then how's he going to pay for it’ *(Robyn)*
	**3**	‘being Māori and not being wealthy you know, some of those places are a bit out of the league yeah, which is not their fault but it's just that you know it's not a comfort place.’ *(Manu)*
	**4**	‘I know all my whānau wait until it's at its worst before they seek support’ *(Tui)*
Fear of discrimination	**5**	‘it’s a fear of judgement. You know, especially with my kids and husband, they’re obviously Māori and the fear that they will be judged as Māori or will not receive the same treatment or access to the same devices that non-Māori might get because they might not appear like they can afford it’ *(Tui)*
Not a welcoming environment	**6**	‘they all have nothing on the wall which shows biculturalism, no posters, no nothing.’ *(Manu)*
	**7**	‘it’s white on white on white’ *(Tui)*
	**8**	‘when a Pākehā person walks in, they are greeted with hello but when they are Māori, never kia ora.’ *(Maia)*
The need for cultural safety	**9**	‘they should be asking themselves how to engage and keep Māori safe.’ *(Brooke)*
By Māori, for Māori services	**10**	‘I think Māori need to be at the table, there needs to be a voice for Māori because it's different, it's not the same as non-Māori.’ *(Tui)*
	**11**	‘I think what I would like, it can't be taught. It’s the mannerisms and I think that creates the atmosphere.’ *(Geoff)*
	**12**	‘with Māori there's more like empathy, understanding, caring.’ Similar to this was a research partner who spoke of an ‘invisible relationship.’ *(Manu)*
	**13**	‘in Māori settings like, you've got so many health services and Kaupapa Māori health services out there for mental health, for addictions, for all those types of things – if you put it there, we'd all know.’ *(Robyn)*
Indigenise current hearing healthcare	**14**	‘I think for me it's just about feeling like you can go into those spaces and that you can hear your own reo.’ *(Tui)*
	**15**	‘just the greetings and farewells’ *(Manu)*
	**16**	‘some of those key cultural concepts like I guess mana and you probably have to have an understanding of tapu and noa.’ (Brooke)
Creation of more TRM assessments	**17**	‘find a starting place where more te reo is implemented. I think that would be a great start.’ *(Rawiri)*
	**18**	‘if te reo was on there then that would be really great and I would really love that.’ *(Moana)*
	**19**	‘I would choose the te reo one to see what it was like, then I’d go off and tell everyone else to go and have a go’ *(Maia)*
TRDMTT as a first step to access	**20**	‘it did feel different, it was you know, I knew it was going to be a Māori screening process, so I was comfortable, there wasn't any barriers to it.’ *(Robyn)*
	**21**	‘it’s Kaupapa Māori and I’m drawn to Kaupapa Māori straight away.’ *(Rawiri)*
	**22**	‘it’s more inviting if it’s run by Māori, for Māori’ *(Maia)*

### Theme one: ‘That’s not for me’

**Figure 1.**
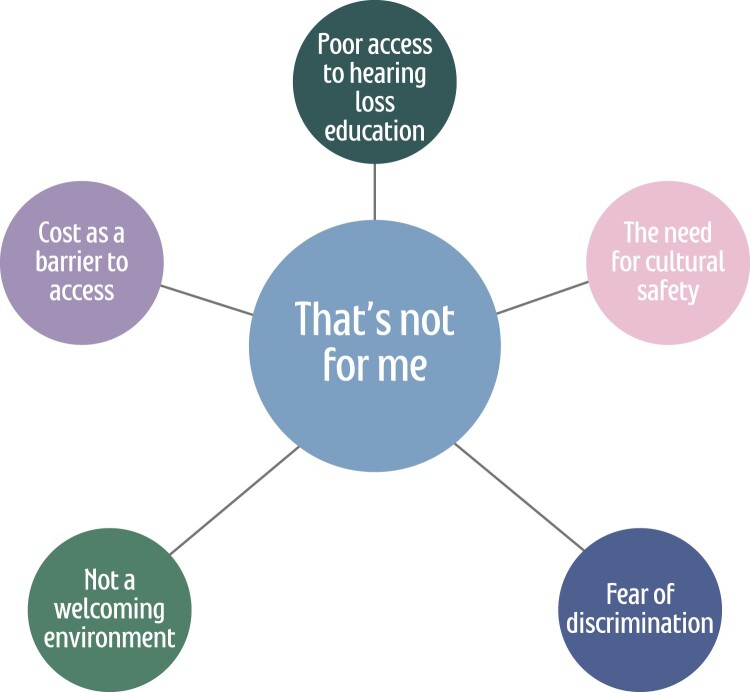
Thematic map one: That’s not for me.

Theme one was developed from an understanding that research partners struggle to see a service that is designed with them in mind. ‘*That’s not for me’,* was said by research partner Tui and it sums up the research partners’ collective experiences and perspectives of ear and hearing healthcare. The theme is broken down into five sub-themes shown in Figure 1.

#### Poor access to hearing loss education

A crucial factor in whether people seek ear and hearing healthcare is their understanding of hearing loss, the symptoms, the impacts it can have and what can be done to help. A concept which was raised by the research partners was the limited education that some kaumātua (Māori elders) receive around what symptoms they are experiencing and when to seek help (see Table 1, quote 1).

#### Cost as a barrier to access

When asked what factors affected their ability to seek or attend ear and hearing healthcare, the research partners often commented on the cost of the services. There was fear associated with the potential cost of rehabilitation devices which stopped them from even seeking an assessment (see Table 1, quote 2). Alongside this was the perception that ear and hearing healthcare is for the wealthy (see Table 1, quote 3).

The inaccessibility of ear and hearing healthcare because of cost has flow on impacts to the overall hauora (health and wellbeing) of the individual and their whānau. There can often be delays in being provided the support and care they require (see Table 1, quote 4) and an increase in risk of negative outcomes associated with a hearing loss.

#### Fear of discrimination

Shame, discomfort, anxiety, hesitancy and fear were all expressed as reasons for not attending or delaying seeking ear and hearing healthcare. Fear because of discrimination they may have experienced in other health service settings, anxiety of entering an unknown and often unwelcoming environment, and alongside these, they were ashamed to receive judgement for their current ear health practices, for being Māori, and for how they looked (see Table 1, quote 5).

#### Not a welcoming environment

Once they navigated barriers and got to the service, research partners experienced an unwelcoming environment (see Table 1, quote 6). The research partners knew that services they accessed had been designed for the majority, with little consideration for how Māori would access and utilise the services (see Table 1, quote 7). They felt the lack of te reo being spoken in the space was a barrier (see Table 1, quote 8). There was a desire to have resources and practices introduced to increase trust and enable an environment where Māori feel welcome and safe.

#### Need for cultural safety

Currently, most ear and hearing healthcare services have not been designed with or for Māori. The research partners’ experiences reflected this, and the need for cultural safety is an important finding of this research. Hearing healthcare practitioners need to question whether the service they are providing is culturally safe (see Table 1, quote 9). The research partners highlighted an understanding of key cultural values, beliefs and practices such as tikanga as well as the process of whakawhanaungatanga (an indigenous process of creating relational connection) as important aspects of cultural safety. If the ear and hearing healthcare workforce was better prepared to communicate with, interact and involve Māori clients in decision-making processes then the service would appear more open and welcoming.

### Theme two: ‘You can’t go wrong with more tikanga’

**Figure 2.**
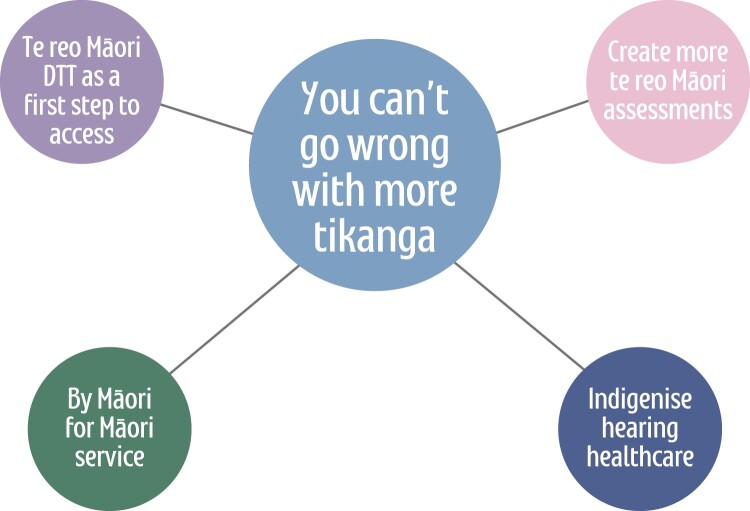
Thematic map two: You can’t go wrong with more tikanga.

The second theme resulted from the research partners’ perspectives on, not only future te reo audiology assessments, but also other steps to create a more equitable ear and hearing healthcare environment for Māori. It is made up of four subthemes, presented in Figure 2.

#### By Māori, for Māori service

Research partners’ idea of ‘by Māori, for Māori’ was multifaceted and included Māori being involved at the base level in the design and delivery of services (see Table 1, quote 10). It was further recognised amongst research partners that there was a lack of Māori working in ear and hearing healthcare services, and there was a consensus that increasing the Māori ear and hearing healthcare workforce would help increase utilisation of the services for them and their whānau (see Table 1, quotes 11 and 12).

They expressed aspirations to see TRMDTT rolled out within existing kaupapa Māori services, as a ‘by Māori, for Māori’ tool (see Table 1, quote 13). Because some research partners already utilised kaupapa Māori services, they suggested the availability of tests in these spaces would enable people to have a hearing screen while they waited for other appointments.

#### Indigenise current ear and hearing healthcare

The responses of our research partners indicate that indigenisation of ear and hearing healthcare will enable wider utilisation of services in Aotearoa than current biomedical approaches to care. The research partners shared a strong desire to see more of themselves and their culture represented in ear and hearing healthcare spaces through the integration of te reo into the environment and use of te ao Māori values beliefs and practices such as tikanga Māori (see Table 1, quotes 14 and 15).

One of these values included whakawhanaungatanga and was viewed as an important aspect of Māori culture that could be applied in ear and hearing healthcare settings to engage the client. A clinician taking as little as five minutes to do a quick introduction of who they are and giving a run through of how the appointment is going to go could help to alleviate fear and make the experience much more enjoyable for the person (see Table 1, quote 16).

#### Creation of more te reo Māori assessments

When questioned about future te reo audiology assessments, research partners found it difficult to conceptualise what these could look like, and their focus was on incorporating te reo in a broader context (see Table 1, quote 17). There was however an underlying desire amongst many of the research partners to be given te reo assessments as an option (see Table 1, quotes 18 and 19).

#### TRMDTT as a first step to access

The experiences of completing a hearing screening tool in te reo were positive. The inclusion of te reo immediately offered a sense of relief and comfort. Because the test only used the digits 0–9, it made the research partners feel more confident about undergoing a hearing screen, regardless of te reo ability (see Table 1, quote 20). Several research partners felt the test is kaupapa Māori, because it embeds a ‘by Māori, for Māori’ screening approach and te reo (see Table 1, quotes 21 and 22). Accordingly, there was a desire to recommend it to friends and whānau. TRMDTT could therefore serve as a tool to help Māori access ear and hearing healthcare.

## Discussion

This study explored eight Māori adults’ perspectives and lived experiences of ear and hearing healthcare services across Te Waipounamu, as well as perspectives on te reo Māori hearing assessments and aspirations for the future of ear and hearing healthcare. The results found that Māori adults face numerous barriers to accessing ear and hearing healthcare, which is consistent with other literature for both ear and hearing healthcare and the wider healthcare system in Aotearoa (Graham and Masters-Awatere 2020; Buckthought et al. 2024). The perspectives shared by the research partners revealed aspirations for future te reo audiological assessments, but also highlighted the current need for culturally safe ear and hearing healthcare and Māori workforce development. Under ‘ko te tuatoru’ of Te Tiriti o Waitangi (Te Tiriti), Māori have the right to ‘Ōritetanga’ which guarantees equitable hearing healthcare outcomes for all. This includes the right to receive hearing healthcare services and assessments in te reo, and the right to a culturally safe, fair and welcoming service.

### Te reo Māori audiological assessments

There is an aspiration to see audiological assessments in te reo. The research partners were excited about having access to a hearing screening tool such as TRMDTT. The use of te reo within a clinical setting provides opportunities to develop appropriate practitioner-client relationships and validates the client as Māori. This is similar whakaaro to those who participated in research looking at te reo use in primary care settings (Pitama 2007; Pitama et al. 2011; Pitama et al. 2014). Actioning te reo revitalisation and indigenisation of hearing healthcare moves the sector closer to being able to fulfill obligations under Te Tiriti by strengthening equity and offering the space for communities to express tino rangatiratanga.

### Taking a kaupapa Māori approach

It is crucial future projects in this space be co-designed with the community. In partnering with Māori, we centre Māori voices and lived realities within research, which leads to interventions and service provision where mātauranga Māori is valued and tino rangatiratanga reinforced (Palmer et al. 2019; Haitana et al. 2020; Smith 2021). By privileging Māori voices and positioning Māori as experts both in their experiences and critiques of the system we can challenge the status quo and improve the current design and delivery of services. Māori-led research is scarce within hearing healthcare, but this research has demonstrated this is required to understand and achieve equity within hearing healthcare.

The value of including tikanga and te reo within hearing healthcare was suggested by Dawson et al. (2024), who concluded there is scope within hearing healthcare to challenge the monopoly of the current biomedical approach and achieve greater efficacy. Research has shown that non-biomedical approaches can more effectively deliver interventions to Indigenous populations through development of trust, reciprocity and shared decision-making (Peiris et al. 2008).

### Cultural safety in ear and hearing healthcare

This research highlighted the current lack of cultural safety within ear and hearing healthcare in Aotearoa. Cultural safety has its origins in nursing where the concept was developed to address the racism experienced by Māori patients when being treated by a largely Pākehā workforce and seeks to achieve better care through acknowledging barriers, considering power relationships, implementing reflective practice, and by allowing the patient to determine whether a clinical encounter is safe (Papps and Ramsden 1996; Curtis et al. 2019). Reflective practice was raised by a research partner as vital to providing culturally safe ear and hearing healthcare services. Also recognised by several research partners was the impact of health professionals who had acknowledged them as Tangata Whenua (Indigenous people of the land) and built rapport through the identification of cultural needs and values.

There is little evidence to suggest that ear and hearing healthcare provides culturally safe services in Aotearoa, and this is reflected in the experiences of the research partners. The application of cultural safety needs to be at both the interface of the individual healthcare provider and the patient, and at the organisational level (Curtis et al. 2019). Within hearing healthcare, it is not only individual clinicians who need to build their knowledge and skills but also the systemic lack of cultural safety will need to be addressed to move towards a more equitable future.

The Pae Ora (Healthy Futures) Act 2022 states that the health sector is responsible for ensuring Māori have access to, and receive, equitable services to achieve equitable health outcomes, as well as providing culturally safe services that are responsive to people’s needs (Ahuriri-Driscoll et al. 2022; Ministry of Health 2022). Audiologists in Aotearoa are self-governed by the New Zealand Audiological Society (NZAS) which means they are not regulated under the Health Practitioners Competence Assurance Act 2003. Under this Act, registered health professionals are bound to standards of cultural competency and safety and clinicians are required to undergo training and regularly demonstrate cultural safety so that the workforce is able to identify and respond to the unique cultural needs of both Māori and their whānau (Ministry of Health 2003, Heke et al. 2019). Currently the NZAS has no standards or guidelines relating to cultural competency and safety for its clinicians. Recent research into the impact of middle ear disease on Māori adults highlights the importance of cultural safety in ear and hearing healthcare services (Buckthought et al. 2024).

Within hearing healthcare service provision and delivery, the sector has obligations under Te Tiriti to promote equitable hearing healthcare and practice guidelines need to be developed which reflect these obligations. In 2022, NZAS added their shared commitment to Te Tiriti into their constitution for the first time as Tangata Tiriti, stating ‘*the Society will enact and comply with policies, practices, and procedures that reflect New Zealand’s commitment to the provisions, spirit, and intent of Te Tiriti o Waitangi’* (New Zealand Audiological Society 2022). A year later, the President of the New Zealand Audiological Society cited the cultural safety of the audiological workforce as its number one priority (Marais 2023). These are steps forward that set a foundation for the establishment of best practice guidelines to support clinicians to develop culturally safe clinical practices.

### Māori health workforce

Increasing the Māori hearing healthcare workforce was identified as a way forward to create culturally safe environments within the confines of the current biomedical approach to hearing healthcare. Cultural safety issues can arise if a workforce is not representative of the population that it serves, and a lack of Māori staff is a proven barrier to Māori accessing healthcare (Jansen et al. 2008; Cram 2014; Palmer et al. 2019). There was a shared whakaaro that the research partners expressed around Māori healthcare workers being perceived as more empathetic and understanding, as they were able to naturally adopt tikanga and processes such as whakawhanaungatanga. The available data suggests Māori make up just 2-3% of the audiology workforce in Aotearoa (Valentine and Fazleen 2018, New Zealand Audiological Society 2024). Literature from other healthcare settings shows a cultural alignment between clinicians and clients influences the acceptability of services, contributes to higher client satisfaction and increases engagement with health services (Maxwell-Crawford 2011; Heke et al. 2019; Wepa and Wilson 2019; Wilson et al. 2022).

Similar needs have been identified overseas. Research into improving speech language therapy for First Nations children around the world echo the need for future services to be co-designed with communities and recommends use of local community health workers as cultural liaisons (Salins et al. 2023). Similarly, a recent scoping review into the ear and hearing health in Aboriginal and Torres Strait Islanders aged over 15 years championed cultural safety and suggested employing Indigenous healthcare workers as a tool to help improve engagement and reduce communication and systemic barriers (Pender et al. 2023).

Designing a healthcare system based solely on the dominant culture’s beliefs and values can lead to significant structural power imbalances (Zambas and Wright 2016). There is no denying this imbalance exists in ear and hearing healthcare, and the call from research partners to see provision of Māori-led research and services reflects a desire to see this corrected. The provision of ‘by Māori, for Māori’ ear and hearing healthcare within existing Kaupapa Māori settings would help to indigenise ear and hearing healthcare. With further resourcing being provided to Māori-led health initiatives working within Kaupapa Māori frameworks these power structures are being challenged in other health fields (Rolleston et al. 2020).

Although this research was conducted in Te Waipounamu, it is relevant to hearing healthcare throughout Aotearoa. This study shows that there is much to be gained by the hearing healthcare sector recognising that what is currently being provided is not culturally safe. At an organisational level, the development of Māori-led hearing healthcare is recommended to address some of the disparities in outcomes. Further exploration of Māori and non-Māori provider perspectives in hearing healthcare is needed.

## Conclusion

The results show that ear and hearing healthcare in Te Waipounamu is often not accessible for Māori adults, who face barriers such as unfamiliar and unwelcoming services where they fear discrimination, prohibitive costs, and culturally unsafe practices. Steps need to be taken to address these barriers if Māori are to achieve the highest attainable level of ear and hearing health. The validation and deployment of TRMDTT is currently underway (see Neame, 2024), however, this research also points to the need for systemic transformation of hearing healthcare in Aotearoa. This research revealed research participants’ aspirations for more Māori-lead and culturally safe ear and hearing health services. The challenge now is to continue working with Māori to realise these aspirations, transform service delivery, and create equitable ear and hearing health outcomes for Māori.
